# Critical Role of the Human ATP-Binding Cassette G1 Transporter in Cardiometabolic Diseases

**DOI:** 10.3390/ijms18091892

**Published:** 2017-09-02

**Authors:** Lise M. Hardy, Eric Frisdal, Wilfried Le Goff

**Affiliations:** Sorbonne Universités, UPMC Univ Paris 06, Inserm, Institute of Cardiometabolism and Nutrition (ICAN), UMR_S1166, Hôpital de la Pitié, F-75013 Paris, France; lise.hardy@inserm.fr (L.M.H.); eric.frisdal@upmc.fr (E.F.)

**Keywords:** ABCG1, triglyceride, high-density lipoprotein, macrophage, lipoprotein lipase, obesity, diabetes, insulin resistance, atherosclerosis, cardiovascular diseases

## Abstract

ATP-binding cassette G1 (ABCG1) is a member of the large family of ABC transporters which are involved in the active transport of many amphiphilic and lipophilic molecules including lipids, drugs or endogenous metabolites. It is now well established that ABCG1 promotes the export of lipids, including cholesterol, phospholipids, sphingomyelin and oxysterols, and plays a key role in the maintenance of tissue lipid homeostasis. Although ABCG1 was initially proposed to mediate cholesterol efflux from macrophages and then to protect against atherosclerosis and cardiovascular diseases (CVD), it becomes now clear that ABCG1 exerts a larger spectrum of actions which are of major importance in cardiometabolic diseases (CMD). Beyond a role in cellular lipid homeostasis, ABCG1 equally participates to glucose and lipid metabolism by controlling the secretion and activity of insulin and lipoprotein lipase. Moreover, there is now a growing body of evidence suggesting that modulation of ABCG1 expression might contribute to the development of diabetes and obesity, which are major risk factors of CVD. In order to provide the current understanding of the action of ABCG1 in CMD, we here reviewed major findings obtained from studies in mice together with data from the genetic and epigenetic analysis of *ABCG1* in the context of CMD.

## 1. Introduction to ABC Transporters and Lipid Metabolism

Adenosine triphosphate (ATP)-Binding Cassette (ABC) transporters belong to a large protein family composed of 48 members in humans, divided into 7 families (A–G) [1]. The ABC transporters mediate the active transport of molecules through membranes by hydrolyzing ATP. Thus, ABC transporters are able to drive transport of different types of molecules, from small inorganic or organic molecules to larger organic compounds, such as hormones, amino acids, ions, vitamins and lipids. Exchange of nutrients and metabolites, which are essential for the maintenance of cell or tissue homeostasis, often occurs against concentration gradients across cellular membranes. Therefore, it is not surprisingly that mutations in genes encoding ABC transporters lead to impaired homeostasis and genetic disorders associated with diseases. More especially, mutations in genes coding for ABC transporters involved in lipid transport are responsible for alterations in cellular lipid homeostasis and cause lipid-related disorders which may be associated with an increased risk of cardiovascular diseases (CVD) [2,3].

Among them, patients carrying mutations in *ABCG5* or *ABCG8* genes exhibit Sitosterolemia [4,5], a pathology characterized by elevated plasma levels of plant sterols due to an exacerbated absorbance of dietary phytosterols from the intestine. Indeed, the ABCG5/ABCG8 dimer promotes the efflux of cholesterol and plant sterols from both hepatocytes to bile and enterocytes to lumen. Patients harboring mutations in either *ABCG5* or *ABCG8* have premature atherosclerosis and an increased risk to develop CVD [6]. In addition, mutations in *ABCA1* cause Tangier disease, a pathology characterized by a quasi-absence of high-density lipoprotein (HDL) and the appearance of lipid depots in tonsils, liver, spleen and lymph nodes [7,8,9]. Indeed, ABCA1 promotes cholesterol and phospholipid efflux to lipid-poor apolipoprotein A-I (ApoA-I) [10] and through this mechanism expression of ABCA1 in liver and intestine is crucial for HDL biogenesis [11]. Cholesterol efflux to HDL from macrophages in the arterial wall is proposed to protect against macrophage lipid accumulation and atherosclerosis development. Thus, mutations in ABCA1 are associated with a premature atherosclerosis and an increased CVD risk [12,13].

Although no genetic disorder caused by mutations in the *ABCG1* gene is reported so far, the ABCG1 transporter draws a special attention since it is described to promote cellular cholesterol efflux to HDL [14,15]. The membrane ABCG1 transporter was initially thought to protect from atherosclerosis and CVD. However, investigation of the role of ABCG1 in mouse models as well as in human studies seems to indicate that the control of cellular lipid homeostasis operated by ABCG1 might play an important role in the wide spectrum of metabolic disorders, including obesity, diabetes and insulin resistance, which are risk factors for atherosclerosis and CVD.

The goal of the present review is therefore to provide a comprehensive view of the action of ABCG1 in lipid metabolism and lipid-related metabolic disorders in the context of cardiometabolic diseases (CMD). In addition to major findings obtained from studies in mice, the present review mainly focusses on the analysis of genetic and epigenetic modulation of *ABCG1* in relation to CMD.

## 2. Structure and Regulation

The human *ABCG1* gene was initially identified as the human homolog of the *Drosophila melanogaster white* gene which encodes a member of the ABC transporter family involved in guanine and tryptophan import, two precursors of Drosophila eye color pigments [16]. In humans, *ABCG1* gene has been mapped to chromosome 21q22.3 [17] and multiple human *ABCG1* transcripts have been detected resulting from different transcription initiation and alternative mRNA splicing [18,19,20]. Similar to others members of the ABC family (ABCA1, ABCG4, ABCG5 and ABCG8), the expression of human ABCG1 is highly stimulated by cholesterol loading and oxysterols [14,21] through the liver X receptors (LXR) pathway. Indeed LXR acts as a cholesterol sensor which is activated by oxysterols (cholesterol oxidative derivatives) when the cholesterol concentration increases in the cell. Induction of ABCG1 expression by LXR agonists likely involves the presence of multiple LXR responsive elements (LXRE) through the *ABCG1* gene [20,22] and appears to only require the LXRα isoform in human macrophages [23]. Analysis of the molecular mechanism indicates that LXR recruitment at the human *ABCG1* locus is facilitated by the G protein pathway suppressor 2 (GPS2) [24]. Although ABCG1 shares common gene expression regulatory pathways with others ABC family members, ABCG1 clearly exhibits a tissue specific expression pattern with high expression levels of ABCG1 found in lung, brain, spleen, adrenal glands, heart and liver [14,17].

**Figure 1:**
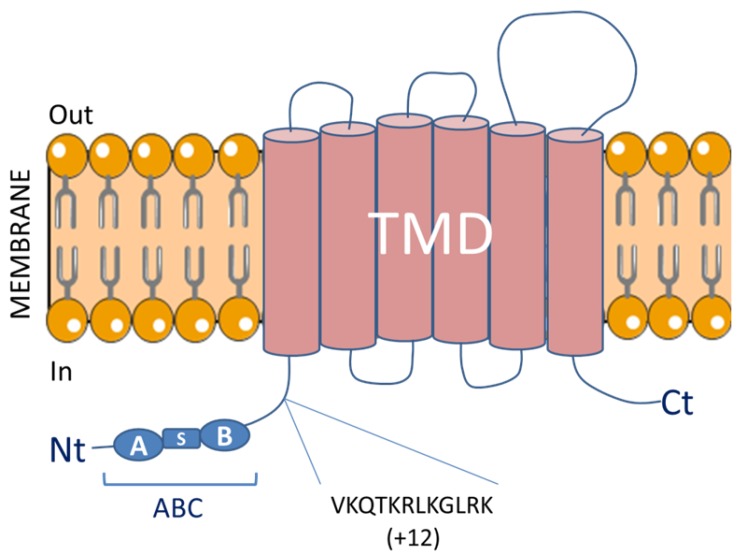
Structure of the membrane ATP-binding cassette G1 (ABCG1) transporter. ABCG1 is a member of the ABC superfamily of transporters and contains one ABC domain containing three highly conserved domains (Walker A (A), Walker B (B) and Signature motif (S)) and one transmembrane domain (TMD) consisting of six membrane-spanning α-helices. The 12 amino acids present in the long ABCG1 isoform (+12AA) are indicated. Functional ABC transporters require two ABCs and two TMDs, therefore, the ABCG1 half-transporter must homodimerize or heterodimerize with others ABC partners in order to be functional. Nt: N-terminus; Ct: C-terminus.

Although discrepancies were initially found in the literature regarding the structure of the *ABCG1* gene [18,19,20], it is now well established that the *ABCG1* gene is composed of 23 exons encoding a protein forming a half transporter with 6 transmembrane spanning domains and a single intracellular nucleotide binding domain (NBD) (Figure 1). This NBD domain contains highly conserved Walker A and Walker B motifs and is required for the binding and the hydrolysis of ATP [25,26] which might provide required energy to transport substrates across the membrane [27]. An alternative splicing at the end of exon 17 led to the generation of a spliced variant of ABCG1 which lacks an internal segment of 12 amino acids in the region between the cytoplasmic domain and the ATP cassette (ABCG1(−12)) [28,29]. Interestingly, the two ABCG1 isoforms, ABCG1(+12) and ABCG1(−12), are expressed in humans whereas mice only express the shorter isoform putting forward the limitations of extrapolating results from mouse models to the human context [30]. Although both isoforms exhibit similar activity, expression of the ABCG1(−12) isoform is roughly two fold more abundant than the ABCG1(+12) isoform in human macrophages and vascular cells [28,29]. Indeed, Gelissen et al. demonstrated that the ABCG1(+12) isoform is more rapidly degraded as the result of the phosphorylation of a serine residue located in the 11 amino acid segment by protein kinase A (PKA) [31]. Others mechanisms contribute to the stability and activity of human ABCG1. Thus, ABCG1 degradation is proposed to be regulated via the ubiquitin proteasome system by the E3 ubiquitin ligases HUWE1 (HECT, UBA, and WWE domain containing 1, E3 ubiquitin protein ligase) and NEDD4-1 (Neural precursor cell-expressed developmentally down regulated gene 4) [32]. Interestingly, ubiquitination and proteasomal degradation of human ABCG1 was suppressed by cholesterol through interaction with a cholesterol recognition/interaction amino acid consensus (CRAC) motif located in the final transmembrane domain of ABCG1 [33,34].

A debate still occurs regarding the cellular localization of ABCG1. Indeed, the use of expression vectors containing the human ABCG1 cDNA sequence fused or not to a tag protein for ABCG1 visualization led to conflicting results with ABCG1 being predominantly detected at the plasma membrane [27,28,35,36,37] or/and in the intracellular compartment [14,38,39,40]. Indeed, localization of human ABCG1 was reported at the cell surface and in the endocytic pathway (early, recycling, late endosomes and lysosomes) as well as in the Golgi apparatus [14,28,35,38,39,40] and ABCG1 was proposed to traffic along the secretory pathway from the endoplasmic reticulum to the cell surface as well as along the endocytic pathway to the late endosomes/lysosomes [41]. The presence of Leu 562 [37] or both Asn 316 and Phe 320 in the highly conserved sequence located between the NBD and the transmembrane domain [42] has been reported to be important for ABCG1 localization at the plasma membrane.

## 3. Functions and Mechanism of Action

The ABC transporters are membrane-bound proteins that mediate the ATP-dependent translocation of many amphiphilic and lipophilic molecules including lipids, drugs or endogenous metabolites. Among them, twenty transporters have been shown to be implicated in lipids, lipid-like or steroids transport [43]. The ABCG1 transporter is a half ABC protein that must dimerize to form an active transporter. It has been reported that human ABCG1 can form homodimers [38] or heterodimers, especially with ABCG4 [26,44]. Although others potential partners for ABCG1 may exist, it is very likely that ABCG1 mainly acts as a homodimer since ABCG1 and ABCG4 are generally spatially separated at the exception of the brain [45]. Human ABCG1 was identified as a lipid transporter able to promote the export of numerous lipid species including cholesterol, phospholipids (phosphatidylcholine), sphingomyelin (SM) and oxysterols (7-ketocholesterol, 7β-hydroxycholesterol and 25-hydroxycholesterol) [36,38,46,47,48,49,50]. However, it appears that ABCG1 exerts a broader array of substrates which is not restricted to lipids as ABCG1 can equally promote export of liposoluble molecules such as vitamin E (α- and γ-tocopherol) [51]. In contrast to ABCA1, ABCG1 promotes cellular cholesterol efflux to high density lipoproteins (HDL_2_ and HDL_3_) but not to lipid-free ApoA-I; the phospholipid content of the acceptor being the major parameter driving ABCG1-mediated cholesterol efflux [38,46]. Importantly, lipidation of ApoA-I by ABCA1 generates nascent or preβ-HDL which may serve as a substrate for ABCG1 indicating that ABCA1 and ABCG1 act sequentially to export cellular cholesterol efflux [38,46]. However, even if HDL is the most efficient lipid acceptor for promoting lipid efflux through ABCG1, the latter may also occur in the presence of non-specific acceptors such as serum albumin although to a lesser degree considering the efflux of cholesterol [36,50].

The mechanism of action of ABCG1 is not yet fully understood and is controversial. A prevailing view is that ABCG1 acts within the endocytic pathway and at the plasma membrane by redistributing membrane cholesterol through the vesicular pathway between endoplasmic reticulum and plasma membrane. The reorganization of membrane cholesterol and SM operated by ABCG1 might increase their accessibility to extracellular acceptors for removal allowing a reduction of lipid rafts formation at the plasma membrane [41,52,53]. However, this process might occur independently of the presence of such acceptors and cholesterol might desorb in a nonspecific manner when ABCG1 is expressed [54].

## 4. Critical Role of ATP-Binding Cassette G1 (ABCG1) in Cardiometabolic Diseases

Identification of a role of ABCG1 in promoting cellular lipid efflux to extracellular acceptors led to propose that ABCG1 may be essential to maintain cell or tissue lipid homeostasis in metabolic contexts associated with major perturbation of lipid homeostasis and metabolism as classically observed in CMD. However, no genetic disease caused by *ABCG1* mutations has been documented and generation of mouse models with targeted disruption of *Abcg1* or expression of human *ABCG1* helped to appreciate the contribution of ABCG1 in physiological and physiopathological situations. Although major finding from studies in mice will be reported here, we will mainly focus on information available when human ABCG1 is explored at both genetic and epigenetic levels which are of major importance for uncovering association of ABCG1 with metabolic traits and providing insights in CMD.

## 5. ABCG1 and Lipid Metabolism and Homeostasis

### 5.1. Major Findings from Studies in Mice

In agreement with a role of ABCG1 in promoting cellular cholesterol efflux, the use of *Abcg1*^−/−^ and human *ABCG1* transgenic mice confirmed that ABCG1 has a critical role in maintaining tissue lipid homeostasis when mice were challenged with a high-fat and high-cholesterol diet [55]. Indeed although neutral lipid accumulation was observed in lung from deficient-*Abcg1* mice on a chow diet [55,56], a massive elevation of the amount of cholesterol ester, triglycerides (TG) and phospholipids was detected in hepatocytes and macrophages from liver and lung when deficient-*Abcg1* mice were fed a high-fat and high-cholesterol diet. By contrast, animals expressing the human *ABCG1* transgene were protected from lipid accumulation in those tissues [55]. Although not the topic of the present review, it is however important to mention here that ABCG1 plays a key role in maintaining lung lipid homeostasis and that impaired ABCG1 expression was reported in pulmonary alveolar proteinosis (PAP) [57]. Indeed, PAP is characterized by deficient surfactant clearance and lipid accumulation in alveolar macrophages which were proposed to result from a defective ABCG1 expression [57,58,59].

The apparent preponderant role of macrophage Abcg1 to export cholesterol was confirmed in peritoneal macrophages from Abcg1-deficient mice which exhibited a reduced cellular cholesterol efflux to HDL but not to lipid free ApoA-I in comparison to peritoneal macrophages from wild-type mice when stimulated with LXR ligands. Despite a role of ABCG1 in promoting cellular cholesterol export to HDL, lack of Abcg1 in mice has no consequence on plasma lipid levels, especially HDL-C. Indeed, both Abcg1-deficient mice and human *ABCG1* transgenic mice fed either a chow diet or a high-fat and high-cholesterol diet exhibited no differences in plasma lipid levels as compared to wild-type mice [55,60,61]. However, Wiersma et al. observed a less pronounced increase in plasma HDL-C in Abcg1 knockout (KO) mice following a 2-week period of high-cholesterol diet or when fed a chow diet after LXR agonist (T0901317) administration, suggesting that LXR activation is required to elicit an effect of Abcg1 on plasma HDL-C levels [62].

### 5.2. Genetic Modulation of Human ABCG1

Development of genome- and epigenome-wide association studies (GWAS and EWAS) provides very useful tools to study the association between genetic and epigenetic variations of the human *ABCG1* gene and metabolic phenotypes. Numerous polymorphisms have been identified in the *ABCG1* locus [63] and several functional single nucleotide polymorphisms (SNP) located in the promoter region as well as in the coding region of the *ABCG1* gene served as genetic markers to explore the association between *ABCG1* and plasma lipid levels and to decipher the role of ABCG1 in CMD. Genotyping of a functional *ABCG1* SNP (rs1378577, T > G) located in the promoter region of the *ABCG1* gene in 109 Japanese men with a coronary artery disease (CAD) did not allow to detect any statistical differences in serum TG, total cholesterol (TC), low-density lipoproteins (LDL)-C and HDL-C according to the *ABCG1* genotype [64]. Similar observations were reported in 609 dyslipidemic white men with a minimum 50% obstruction of a major coronary artery in regression growth evaluation satin study (REGRESS) when two different SNPs located in the *ABCG1* promoter (rs1378577, T > G and rs1893590, A > C) were studied [65]. Indeed, these two *ABCG1* SNPs were associated with neither plasma lipid concentrations (TC, LDL-C, HDL-C, TG and Lipoprotein(a)) nor circulating cholesterol ester transfer protein (CETP) concentrations. Nevertheless in this study, the authors brought to light an unexpected association between both *ABCG1* SNPs and plasma lipoprotein lipase (LPL) activity independently of any effect on circulating LPL mass [65]. However the absence of association between the two SNPs and TG levels observed in REGRESS (plasma TG levels < 3.5 g/L) suggests that modulation of LPL activity by ABCG1 might only alter circulating TG concentrations in subjects exhibiting fasting or postprandial hypertriglyceridemia. The absence of any association between *ABCG1* promoter SNPs (rs2234714, −768G > A; and rs57137919, −367G > A) and plasma lipids levels (HDL-C, LDL-C, TG and TC) was equally reported in 1021 patients with CAD and 1013 unaffected control subjects in a Chinese Han population [66]. Genotyping of the rs57137919 in 200 Chinese healthy volunteers led to similar conclusions [67]. Association studies in larger cohorts enabled detection of an association between *ABCG1* genotype and plasma HDL-C levels. Indeed, genotyping of 10,237 individuals from the Copenhagen city heart study (CCHS) for 16 *ABCG1* SNPs identified two rare variants (rs140837853, C > T and rs56140811, C > T) located in the promoter region and exon 9 of the *ABCG1* gene that were associated with modest reductions in HDL-C (7% and 3%, respectively); the carriers of the rare alleles exhibited lower HDL-C levels than the non-carriers [68]. However except for HDL-C, none of those *ABCG1* variants were associated with plasma lipids, lipoproteins and apolipoproteins. Moreover, a high-density genotyping array containing SNPs from HDL-C candidates provided suggestive evidence for an association of an intronic *ABCG1* SNP (rs914189, C > G) with HDL-C concentrations in a meta-analysis comprising 7857 individuals [69]. Interestingly, Abellán et al. reported that the *ABCG1* SNP rs1893590 (A > C) was associated with plasma HDL-C levels in the postprandial state while no association was detected with postprandial TC or LDL-C [70]. Indeed, in 1473 Spanish subjects from the population-based Hortega study, the carriers of the rare C allele (CC + AC) were significantly associated with higher postprandial HDL-C concentrations 3 h after lunch as compared to individuals homozygous for the frequent A allele (genotype AA) [70]. Strikingly, this association was only observed in women but not in men suggesting a gender-specific effect of the *ABCG1* genotype on postprandial HDL-C levels. Since people in Western countries spend most of the day in the postprandial state, this study provides new insights regarding the potential contribution of ABCG1 in determining circulating levels of HDL-C, which are firmly reported to be inversely associated to CAD [71]. However, the diet intake appears to be essential in the effect of ABCG1 on plasma HDL-C levels. Indeed, the association between rs1893590 and postprandial HDL-C disappeared after adjustment for caloric intake or lipid consumption [70] whereas an association with the *ABCG1* SNP rs1044317 (G > A, 3’UTR region) with variations in fasting HDL-C concentrations was only observed in subjects with a high polyunsaturated fatty acid (PUFA) intake (>13.6 g/day) [72]. A similar diet-interaction was reported regarding the association of the *ABCG1* SNP rs4148102 (G > A, intronic region) with TC and LDL-C in this latter study [72]. To note that the body mass index (BMI) and age equally interact in the relation between *ABCG1* polymorphisms and plasma HDL-C. Thus, the association between the *ABCG1* SNP rs1893590 (A > C) with HDL-C in an asymptomatic Brazilian population (654 normolipidemic volunteers) was only detectable for individuals under 60 years or BMI < 25 kg/m^2^ with homozygous AA subjects displaying higher plasma HDL-C levels than carriers of the C allele (AC + CC) [73]. Finally, rare *ABCG1* SNPs were reported to be associated with plasma TC (rs170444, A > G) and TG levels (rs170444, A > G and rs8126601, G > A) in a large-scale meta-analysis across 32 studies (66,240 individuals of European ancestry) [74]. However no association between *ABCG1* SNPs (rs1378577, −134 T > G and rs1893590, −204 A > C) with circulating lipids levels was observed in a population of morbidly obese patients (BMI > 40 kg/m^2^, *n* = 1320) [75].

### 5.3. Epigenetic Modulation of Human ABCG1

Epigenetic mechanisms such as DNA methylation might contribute to the determination of circulating lipid concentrations in humans [76]. In this context, recent evidence indicates that epigenetic marks within the *ABCG1* gene including cytosine guanine dinucleotide (CpG) site methylation are associated with blood lipid levels. Genome-wide DNA methylation analysis in whole blood samples of 1776 subjects from the general population of the cooperative health research in the region of Augsburg (KORA) identified CpG sites located in *ABCG1* associated with HDL-C and TG levels [77]. Indeed, cg27243685 and cg07397296 were strongly associated with TG levels whereas cg06500161 was associated in opposite directions with plasma HDL-C and TG levels. Interestingly, association of *ABCG1* DNA methylation with TG levels was also found in adipose tissue, a tissue in which *ABCG1* gene was expressed. Methylation of *ABCG1* at cg06500161 was negatively associated with *ABCG1* mRNA levels indicating that this CpG site was functional in repressing *ABCG1* expression [77,78,79]. Moreover, functional analysis of cg06500161 by electrophoretic mobility shift assay revealed a higher binding of a protein complex for the unmethylated status of the CpG site as compared to the methylated status, suggesting that cg06500161 modulates transcriptional activity of the *ABCG1* promoter. In agreement with those observations, *ABCG1* mRNA levels showed a strong positive association with HDL-C and a negative association with TG levels [77].

The association of DNA methylation at *ABCG1* locus in whole blood DNA with plasma TG and HDL-C levels was replicated in several studies recapitulated in Table 1 which were conducted in the Botnia prospective study (cg06500161, TG, *n* = 258) [80], the Rotterdam study (cg06500161, TG and HDL-C, *n* = 1485) [78], the Framingham heart study (FHS) and prospective investigation of the vasculature in uppsala seniors study (PIVUS) (cg06500161, TG and HDL-C, cg27243685 and cg01176028, TG, *n* = 2036) [79] and in Canadian familial hypercholesterolemia patients (CpGC3, TG, HDL-C and TC, *n* = 98) [81]. In addition, Lai et al. reported that methylation of the *ABCG1* CpG site (cg06500161) in blood CD4^+^ T cells from 979 subjects of the genetics of lipid lowering drugs and diet network (GOLDN) study was significantly associated with postprandial hypertriglyceridemia (PPHT) after consumption of a high-fat test meal [82]. Indeed, an increased methylation at *ABCG1* was correlated with an increased PPHT response. However cg06500161 was equally associated with fasting TG, then the effect on PPHT disappeared after adjustment for baseline fasting TG levels. Finally, beyond TG and HDL-C, EWAS between DNA methylation and lipidomic traits in human blood revealed an association between methylation at cg06500161 and SM, phosphatidylcholine, lysophosphatidylcholine and monoacylglycerol [79,83].

Taken together, the use of polymorphisms in the *ABCG1* gene as genetic markers in association studies appears poorly informative in order to reveal a potential impact of ABGC1 on lipid metabolism. However, the results from DNA methylation studies univocally revealed a strong association of ABCG1 with circulating TG levels and in a lesser extend those of HDL-C. The inverse association between methylation at cg06500161 and HDL-C is coherent with the largely described role of ABCG1 in promoting cellular cholesterol efflux to HDL and suggests that DNA methylation of *ABCG1* in key tissues controlling HDL metabolism (liver or intestine) might contribute to the determination of circulating HDL-C levels. In addition, the strong positive relationship existing between DNA methylation at the *ABCG1* locus and circulating TG levels is in agreement with the role of ABCG1 in controlling the bioavailability and activity of LPL, a key enzyme in TG metabolism [65]. However, it must be kept in mind that although DNA methylation may modulate circulating lipid concentrations, an inverse relationship may equally occur. Indeed, Dekkers et al. demonstrated that blood lipids influenced DNA methylation with methylation of *ABCG1* CpG sites (cg06500161 and cg27243685) being modulated by TG or HDL-C [84].

## 6. ABCG1: Role in Atherosclerosis and Cardiovascular Diseases

Cardiovascular diseases are one of the major causes of mortality and morbidity worldwide and are frequently associated to atherosclerosis, a physiopathological process leading to lipid accumulation, mainly in macrophages in the arterial wall. Epidemiological studies have largely reported that plasma LDL-C levels are positively associated to CAD whereas an inverse relationship exists with those of HDL-C [71].

### 6.1. Major Findings from Studies in Mice

Because of the important role of Abcg1 in maintaining tissue lipid homeostasis, especially in macrophages [55], Abcg1 was proposed to protect from atherosclerosis development by promoting cholesterol efflux to HDL from arterial macrophages. In addition, ABCG1 was reported to protect macrophage from apoptosis induced by oxidized LDL [85] as well as against endothelial dysfunction [48] and activation [86] in mice, which are key features of atherosclerosis. However investigation of the role of Abcg1 in atherosclerosis in animal models led to conflicting results. Indeed, the expression of human ABCG1 in a rabbit fed a high-cholesterol diet [87] or in *Ldlr*^−/−^ [88] or *Apoe*^−/−^ [61] mice fed a high-fat and high-cholesterol diet showed either attenuation or increased or no effect on atherosclerosis, respectively. In addition, Abcg1 deficiency in mice under high-fat and high-cholesterol feeding was either protective or deleterious against atherosclerosis in wild-type mice [60] or in *Apoe*^−/−^ mice [89], respectively. In order to evaluate the specific contribution of macrophage Abcg1 in atherosclerosis development, bone marrow transfer (BMT) from donor *Abcg1*^−/−^ mice into recipient *Ldlr*^−/−^ or *Apoe*^−/−^ mice fed a high-fat and/or a high-cholesterol diet was simultaneously conducted in three different laboratories [89,90,91,92,93,94]. However, consistent with what was observed for modulation of whole body Abcg1 expression, those studies led to contrasting results which did not resolve the role of macrophage Abcg1 in atherosclerosis. In an attempt to reconcile the results obtained in *Ldlr*^−/−^ mice, Meurs et al. proposed that the effect of Abcg1 deficiency on atherosclerosis depends on the stage of the disease [95]. Thus, Abcg1 would be protective in early lesions likely by promoting macrophage cholesterol efflux and reducing foam cell formation whereas Abcg1 would be deleterious in advanced lesions by protecting macrophage from apoptosis through the export of cellular oxysterols. However further investigations are needed to validate this model and to elucidate the whole spectrum of action of Abcg1 in the process of atherogenesis in which Abcg1 appears to exert a more complex role that initially thought.

Finally, since Abcg1 was demonstrated to cooperate with Abca1 in promoting cholesterol efflux from macrophages [38,46], Abca1/Abcg1 double KO mice have been generated in order to determine if the impact of macrophage Abcg1 deficiency in atherogenesis could be unmasked when Abca1 is absent. A much more pronounced reduction of cholesterol efflux to HDL was observed in double Abca1/Abcg1-deficient macrophages in comparison to single KO mice whereas cholesterol efflux to ApoA-I was not affected [92,96]. However, similar to what was observed in single Abcg1-deficiency, BMT from donor *Abca1*^−/−^*Abcg1*^−/−^ mice into recipient *Ldlr*^−/−^ mice led to conflicting results and did not help to elucidate the role of macrophage Abcg1 in atherosclerosis [92,93]. Indeed, an exacerbated size of atherosclerotic lesions was detected in *Ldlr*^−/−^ mice transplanted with *Abca1*^−/−^*Abcg1*^−/−^ BM fed a high-cholesterol diet in comparison to single KO animals [92] whereas an independent study showed no difference in lesion size between *Ldlr*^−/−^ mice transplanted with either *Abca1*^−/−^*Abcg1*^−/−^ or *Abcg1*^−/−^ BM when animals were fed a high-fat and high-cholesterol diet [93].

### 6.2. Genetic Modulation of Human ABCG1

Whole genome expression arrays performed in circulating monocytes (CD14+ cells) from young male with premature familial CAD and control individuals (*n* = 22 in each group) revealed that ABCG1 expression was down-regulated in CAD patients suggesting that ABCG1 might be associated with early atherosclerosis [97]. However, association studies between *ABCG1* SNPs and CAD or atherosclerosis led to conflicting results. Indeed, analysis of the effect of the functional *ABCG1* SNP (rs1378577, T > G) on CAD severity in 109 Japanese men with CAD indicated that individuals homozygous for the T allele (TT) exhibited an increased risk of multi-vessel disease compared with single-vessel disease which was independent of any effect on plasma lipid levels [64]. A protective role of ABCG1 regarding CAD was also reported by Schou et al. who identified two *ABCG1* variants associated with an increased risk for myocardial infarction (MI) and ischemic heart disease (IHD) (rs72542412, −376 C > T and S630L) and one *ABCG1* variant only associated with MI (rs138515663, −311 T > A) in 10,237 individuals from theCCHS [68]. It is worthy to note that those very rare variants were not associated with plasma HDL-C levels. Conversely, *ABCG1* SNPs associated with circulating HDL-C concentrations were not found associated to either MI or IHD indicating that the effect of *ABGC1* variation on the risk of ischemic vascular disease was not the result of any modulation of HDL-C. However, genotyping of two *ABCG1* SNPs located in the ABCG1 promoter (rs1378577, −134 T > G and rs1893590, −204 A > C) in REGRESS indicated that those SNPs were not associated with angiographic parameters (minimum segment diameter and mean obstruction diameter) in individuals (*n* = 609) developing atherosclerosis [65]. On the contrary, analysis of the association of four *ABCG1* polymorphisms, three located in the promoter (rs2234714, −768 G > A; rs2234715, A > G and rs57137919, −367 G > A) and one in the 3’UTR region (rs1044317, G>A) in patients with CAD (*n* = 1021) and unaffected control subjects (*n* = 1013) in a Chinese Han population concluded to a deleterious role of ABCG1 regarding CAD [66]. Indeed, a significant difference in allele frequency and genotype distribution of both rs2234714 and rs57137919 was observed between CAD patients and control subjects with the frequency of the less minor allele being lower in the CAD group (with MI or not) than in controls. Thus, those polymorphisms showed a decreased risk for CAD (rs2234714 and rs57137919) and MI (rs57137919) and led to identification of a GAGA haplotype (rs2234714, rs2234715, rs1044317 and rs57137919) including the more frequent allele of each SNP that was associated with an increased risk of CAD or MI [66]. In addition, rs57137919 was shown to be associated with angiographic severity of CAD (multi-vessel versus single vessel). Interestingly, those SNPs were not in apparent linkage disequilibrium and were not associated with blood lipid concentrations. In order to elucidate the mechanism underlying the deleterious effect of the *ABCG1* SNP rs57137919 in CAD, the same group extended their investigation by carrying out functional experiments in 200 Chinese healthy volunteers [67]. In this study, human monocyte-derived macrophages from individuals carrying the rare AA genotype for the rs57137919 displayed reduced ABCG1 expression, impaired oxidized NBD-cholesterol efflux to HDL and increased apoptosis as compared to macrophages from individuals carrying the frequent GG genotype. In addition, mRNA levels of proapoptotic Bcl-2 related ovarian killer (*BOK*) and BH3 interacting domain death agonist (*BID*) genes from macrophages with the AA genotype were more elevated than those from macrophages with the GG genotype following exposure with oxidized LDL. Those results suggest that the reduced risk of developing atherosclerosis observed in individuals carrying the less frequent rs57137919 A allele could result from an increased macrophage apoptosis, thereby supporting the model proposed by Meurs et al. from studies in mice about the role of Abcg1 in atherosclerosis [95]. Finally, the association of two functional *ABCG1* SNPs (rs1378577, T > G and rs571137919, G > A) with the risk of ischemic stroke in a case-control study including 389 ischemic stroke patients and 380 healthy subjects in the Chinese Han population provided additional mechanistic insights about the deleterious effect of ABCG1 [98]. Indeed, Li et al. demonstrated that the frequency of homozygosity for the rare alleles (GG and AA) was lower in patients as compared to control individuals in a subgroup of hypertriglyceridemia whereas genotypic distribution was similar in patients and controls when the whole population was studied. In addition, both rs1378577 and rs571137919 were associated with a reduced risk of developing ischemic stroke in hypertriglyceridemic individuals. Although the sample size was here relatively low, this study supports the mechanism proposed by Olivier et al. through which ABCG1 could exert a deleterious role in metabolic situations associated with high levels of circulating TG-rich lipoproteins [65]. Indeed, Olivier et al. demonstrated that ABCG1 controls LPL activity and promotes lipid accumulation in macrophages in a presence of TG-rich lipoproteins and, therefore, might contribute to foam cell formation and atherosclerosis development in hypertriglyceridemic individuals.

### 6.3. Epigenetic Modulation of Human ABCG1

Analysis of the relationship between *ABCG1* promoter methylation and coronary heart disease (CHD) was investigated in a small group of 85 CHD patients and 54 participants without CHD in the Han Chinese population [99]. In this study, *ABCG1* promoter hypermethylation was frequently observed in patients with CHD (90.5%) but not in individuals without CHD (29.6%). Further analysis indicated that hypermethylation of the *ABCG1* promoter gene was associated with an increased risk of CHD after adjustment for age, gender, smoking, lipid levels, hypertension and diabetes. In support of this preliminary study, analysis of genome-wide DNA methylation in whole blood in subjects (*n* = 1776) from the general population of the KORA study indicated that the *ABCG1* CpG site cg06500161 was associated with MI [77]. This effect was independent of the association of cg06500161 with HDL-C and TG in this cohort. However, this result must be interpreted with caution since only 60 individuals with previous hospitalized MI were included in this analysis. A very recent study reported that whole blood methylation at the *ABCG1* CpG site cg27243685 (5’UTR region of *ABCG1*) was significantly associated with CHD in a meta-analysis of theFHS and PIVUS (2306 participants, number of CHD events = 193). Altogether, this study highlighted a pathway linking hypermethylation of *ABCG1* at cg27243685 with reduced *ABCG1* expression, higher TG, lower HDL-C and increased risk for CHD [79]. Measurement of blood DNA methylation in Canadian Familial Hypercholesterolemia patients did not allow to establish any association between methylation levels at a single *ABCG1* CpG site (CpGC3) and a prior history of CAD in a small subset of individuals (Non-CAD (*n* = 22) vs CAD (*n* = 22)) [81].

To conclude, association studies in humans using methylation marks at the *ABCG1* locus support a protective role for ABCG1 in CAD in the general population with methylation levels at *ABCG1* CpG sites being associated with an increased risk of CHD and MI. However, this effect is independent of any effect on blood lipid concentrations. Thus, modulation of DNA methylation by environmental factors such as smoking and nutrition might play an important role in the contribution of ABCG1 in CAD. Nevertheless, genome-wide DNA methylation analysis in large cohorts of CAD patients or in case-control studies for CAD will be required to conclude on the impact of methylation at the *ABCG1* locus on CAD. By contrast, results from the genotyping of *ABCG1* SNPs in CAD patients or in case-control studies for CAD support the contention that ABCG1 might exert a pro-atherogenic effect locally in the arterial wall independently of any modulation of circulating HDL-C or TG levels [100]. Such a deleterious role could result from the critical contribution of human ABCG1 in reducing macrophage apoptosis through efflux of oxysterol [50,101] and in promoting macrophage lipid accumulation through modulation of LPL activity in a TG-rich metabolic context [65]. In contrast to what is proposed in mice, human ABCG1 does not promote macrophage cholesterol efflux to HDL [52] and therefore is not expected to attenuate foam cell formation in early atherosclerosis lesions. However, ABCG1 could protect from atherosclerosis by preserving vascular endothelium from dietary cholesterol-induced dysfunction [48,102].

## 7. ABCG1: Role in Diabetes and Insulin Resistance

The presence of diabetes and insulin resistance (IR) is frequently observed in CMD. In addition, diabetes is growing worldwide because of the emergence of obesity and is a risk factor for accelerated atherosclerosis and CAD. The loss of insulin signaling or secretion as well as the loss of glycemic control are critical features that characterize type 2 diabetes (T2D).

### 7.1. Major Findings from Studies in Mice

Macrophages from diabetic mice (*db/db* mice or treated with streptozotocin) showed a decrease of Abcg1 expression and an impaired cellular cholesterol efflux to HDL [103,104]. As a consequence, an increased accumulation of esterified cholesterol was observed in diabetic *db/db* macrophages [103]. Interestingly, Mauldin et al. also demonstrated that elevated glucose led to a repression of Abcg1 expression in mouse macrophages, suggesting that hyperglycemia in diabetes could contribute to foam cell formation and accelerated atherosclerosis through inhibition of macrophage Abcg1-mediated cholesterol efflux. Advanced glycation end products (AGE), which are generated by non-enzymatic glycation and oxidation of proteins and lipids, accumulate in diabetes. Treatment of macrophages with AGEs led to a decreased ABCG1 expression through a peroxisome proliferator-activated receptor (PPAR)γ-dependent mechanism and a reduction of cholesterol efflux to HDL. Generation of mice deficient for the receptor of AGEs (RAGE/AGER) revealed that macrophage reverse cholesterol transport to feces was reduced in diabetic mice through RAGE [104]. Moreover, Nagelin et al. reported that macrophage Abcg1 expression was significantly suppressed by PUFAs such as linoleic or arachidonic acids which frequently accumulate in diabetes and IR [105]. Interestingly, those PUFAs are ligands for 12/15-lipoxygenase (12/15LO) for producing hydroperoxy FA 12S- and 15S-hydroxyeicosatetranoic acids (12SHETE/15SHETE) and 13S-hydroxyoctadecadienoic acid (13SHODE) and 12/15LO was reported to play a role in diabetes and atherosclerosis. Genetic manipulation of 12/15LO in mice indicated that 12/15 LO regulates the degradation of Abcg1 through p38- and c-Jun N-terminal kinase 2 (JNK2)-dependent pathways and macrophage cholesterol efflux to HDL [106].

Beyond macrophages, Abcg1 was demonstrated to play a major role in regulation of subcellular cholesterol distribution in mouse pancreatic β cells [107]. Indeed, loss of Abcg1 in β cells reduced granule cholesterol content and altered granule morphology leading to an impaired insulin secretion. Thus, *Abcg1*^−/−^ mice fed a chow diet exhibited an impaired glucose tolerance and insulin secretion as compared to *Abcg1*^+/+^ animals whereas insulin sensitivity was not altered. The relevance of those findings was strengthened by the observation that islet Abcg1 expression was decreased in hyperglycemic diabetic *db/db* mice and restored by the antidiabetic thiazolidinedione (PPARγ agonist). However, although insulin secretion was equally reduced in *Abcg1*^−/−^ mice fed a chow diet in the work from Kruit et al., glucose tolerance was not affected in *Abcg1*^−/−^ mice when compared to *Abcg1*^+/+^ animals [108]. On the contrary, Abcg1-deficient mice fed a high fat diet were protected from glucose intolerance in comparison to their wild-type littermates [109]. Taken together, a large body of evidence suggests that Abcg1 could play a role in diabetes as well as in accelerated atherosclerosis associated to diabetes through modulation of insulin secretion and foam cell formation, respectively. However, it is still difficult to evaluate the impact of the impaired insulin secretion consecutive to Abcg1 deficiency in diabetes since glucose intolerance was not a common feature in *Abcg1*^−/−^ mice. In addition, although macrophage Abcg1 expression was consistently found reduced in diabetic mice or in response to stimuli relevant to diabetes (PUFA, glucose, AGEs), the impact of such a reduction on macrophage cholesterol efflux and potentially atherogenesis was not evaluated directly in experimental models in which Abcg1 expression may be manipulated (*Abcg1* KO or *Abcg1* KD). Additional investigations in appropriate mouse models are therefore required in order to evaluate the overall physiopathological consequences of the reduced Abcg1 expression in diabetes.

### 7.2. Genetic Modulation of Human ABCG1

Similar to what was observed in diabetic mice, expression of macrophage ABCG1 was reduced in patients with T2D as compared to control subjects [110]. Indeed, blood monocyte-derived macrophages from T2D patients displayed a decreased cholesterol efflux to HDL, but not to ApoA-I, and an increased cholesterol accumulation relative to control individuals. Interestingly, treatment with a LXR agonist induced ABCG1 expression and attenuated cholesterol accumulation in macrophages from T2D patients [110]. A reduction of *ABCG1* expression was also reported in peripheral blood mononuclear cells from Iranian patients with Metabolic Syndrome exhibiting high fasting glycemia in comparison to the control group [111]. Consistent with studies in murine cells, high glucose and PUFAs repressed ABCG1 expression in human macrophages [112,113] whereas a conjugated linoleic acid isomer (trans-9, trans-11-CLA) was reported to activate ABCG1 expression through sterol regulatory element-binding protein (SREBP)-1c [114]. In order to determine whether genetic variation in *ABCG1* may predict T2D in the general population, Schou et al. genotyped several *ABCG1* SNPs in 40,600 individuals from the CCHS [115]. However, none of the 14 *ABCG1* SNPs tested was associated with an increased risk of T2D. Finally, analysis of two functional *ABCG1* SNPs (rs1378577, −134 T > G and rs1893590, −204 A > C in the promoter region) in 1320 French morbidly obese patients (BMI > 40 kg/m^2^) failed to detect any association between the 2 SNPs and homeostatic model assessment of insulin resistance (HOMA-IR) with or without adjustment for BMI [75].

### 7.3. Epigenetic Modulation of Human ABCG1

Although analysis of genetic variations in *ABCG1* failed to report any relationship between ABCG1 and diabetes, numerous studies highlighted a link between epigenetic modulation of *ABCG1* and IR and diabetes. Hidalgo et al. first reported that DNA methylation at the *ABCG1* locus was significantly associated with insulin and HOMA-IR [116]. Indeed, EWAS conducted in blood CD4^+^ T cells from 837 non-diabetic individuals in the GOLDN study identified that methylation at two *ABCG1* CpG site (cg06500161 and cg01881899) was associated with fasting insulin and HOMA-IR. This observation was replicated and enriched in a recent EWAS performed in whole blood samples from 1440 non-diabetic individuals of the KORA F4 study [117]. In this study, the *ABCG1* CpG site cg06500161 was associated with reduced *ABCG1* expression and positively with fasting glucose, 2-h glucose, fasting insulin, 2-h insulin, glycated haemoglobin (HbA1c) and HOMA-IR, effects persisting after adjustment with BMI. Analysis of DNA methylation in a nested case-control study in Indian Asians and Europeans with incident T2D from the 8-year follow-up of 25,372 participants in the London life sciences prospective population (LOLIPOP) revealed that methylation at the *ABCG1* locus (cg06500161) was associated with future T2D incidence [118]. Indeed, the relative risk for incident T2D per 1% increase in methylation was 1.09 (95% CI: 1.07–1.11) at the *ABCG1* methylation marker. A similar risk for future T2D (OR = 1.09, 95% CI: 1.02–1.16) was reported by Dayeh et al. when DNA methylation at the *ABCG1* locus cg06500161 was conducted in blood DNA from 258 non-diabetic individuals from the Botnia prospective study [80]. In those two studies, cg06500161 was positively associated with diabetes-related traits including fasting glucose, HbA1c, fasting insulin and HOMA-IR. Interestingly, DNA methylation at cg06500161 was increased in blood and adipose tissue from subjects with T2D versus non-diabetic subjects [80]. Additional investigations in 850 pedigreed Mexican-American individuals from 39 families in the San Antonio family heart study (SAFHS) confirmed that methylation in *ABCG1* is a determinant of T2D in this population at high risk of developing T2D [119]. Thus, even after adjustment with BMI, cg06500161 was found significantly associated with fasting blood glucose, HOMA-IR and T2D. Further exploration was conducted in this population in order to evaluate genetic and epigenetic associations with the hypertriglyceridemic waist (HTGW) phenotype (high waist circumference (≥95 cm in men and ≥80 cm in women) combined with high serum TG concentration (≥2 mmol/L in men and ≥1.5 mmol/L in women)), as a marker of T2D and CVD [120]. Whereas GWAS detected no SNP in association with HTGW, EWAS identified that the *ABCG1* CpG site (cg06500161) was associated with HTGW and T2D, the association with T2D being significantly explained in part through HTGW.

As a whole, genetic and epigenetic studies of *ABCG1* in T2D and IR univocally demonstrated that genetic modulation of *ABCG1* was without effect on T2D and associated traits whereas methylation marks at the *ABCG1* locus were consistently associated with T2D as well as the risk to develop T2D. Indeed, methylation at *ABCG1* CpG sites was positively associated with all the diabetes-related traits including fasting glucose, fasting insulin, HbA1c and HOMA-IR suggesting that epigenetic regulation of ABCG1 expression by environmental exposure is a critical event in the prevalence and incidence of diabetes.

## 8. ABCG1: Role in Obesity and Weight Gain

### 8.1. Major Findings from Studies in Mice

No difference in body weight was reported in Abcg1-deficent mice or mice expressing human ABCG1 when fed a chow or a high-cholesterol diet supplemented with or without fat [55,60,61,88]. However, genome-wide screens in drosophila and mice led to the identification of *Abcg1* as a candidate for TG storage and obesity [109]. Indeed, Buchman et al. first reported that targeted disruption of *Abcg1* protected mice against diet-induced obesity (DIO) by reducing body weight gain and adipose tissue mass. In addition, *Abcg1*^−/−^ exhibited a markedly decrease of the adipocyte size, an elevation of total energy expenditure and a reduction of food intake as compared to wild-type littermates. The possible underlying mechanism was afterward elucidated by Frisdal et al. who provided evidence that adipocyte Abcg1 exerts a major role in adiposity and fat mass growth [75]. Indeed, Abcg1 ensured the optimal LPL-mediated TG hydrolysis in mouse adipocytes through promoting SM export which contributed to a concomitant induction of adipogenesis under the control of PPARγ and an increase of de novo TG synthesis and storage. Validation of the role of Abcg1 in adipocyte maturation was achieved by the local RNAi-silencing of Abcg1 expression in adipose tissue from mice fed a high-fat diet which led to a rapid decrease of adiposity and fat mass growth. Whereas an increased expression of adipose perilipin (*Plin*), *Pparγ*, and hormone-sensitive lipase (*hsl*) was observed in adipose tissue from male *Abcg1*^−/−^ mice as compared to wild-type littermates upon a high-fat diet [109], the expression of those genes was markedly reduced in *Abcg1*-deficient adipocytes or in *Abcg1* knock down adipose tissue from mice fed a high-fat diet [75]. Those contrasting results clearly highlight that the expression of Abcg1 in cells others than adipocytes contributes, likely to an opposite way, to adipogenesis and adipocyte maturation. However, expression of Abcg1 appears equally critical in controlling inflammation in adipose tissue (AT). Indeed, Abcg1-mediated cholesterol efflux to HDL in mouse adipocyte generated an anti-inflammatory response that reduced expression of pro-inflammatory cytokines and chemotactic factors [121]. Thus, inhibition of Abcg1 expression in mouse adipocytes led to a reduction of plasma cholesterol content, a disruption of lipid rafts and an inhibition of the translocation of NADPH oxidase 4 into lipid rafts. As a consequence, reactive oxygen species (ROS) generation and inflammatory response induced by palmitate was abrogated following silencing of Abcg1. Moreover, BMT from donor *Abcg1*^−/−^ or *Abcg1*^+/+^ mice into recipient C57BL/6 mice fed a high-fat diet revealed that *Abcg1*^−/−^ adipose tissue macrophages (ATM) from obese mice were enriched in cholesterol as compared to *Abcg1*^+/+^ ATM [122]. Analysis of ATM activation indicated that alternatively activated (M2) macrophages were more abundant in *Abcg1*^−/−^ ATM and that this effect could likely result from an impaired M2 macrophage chemotaxis due to Abcg1 deficiency. However, deletion of Abcg1 in myeloid cells in this study did not improve obesity or glucose intolerance [122]. Intriguingly, whereas Abcg1 expression was consistently increased in AT from obese mice as compared to lean mice [109,123], a further elevation of Abcg1 in epididymal AT was observed in *db/db* mice following caloric restriction [123]. The increase of Abcg1 expression in AT following caloric restriction was thought to result from an induction of Abcg1 in ATM and more especially in F4/80 expressing cells. Thus, the effect of myeloid Abcg1 on the regulation of ATM cholesterol content and M2 abundance in AT from obese mice was exacerbated by caloric restriction [122]. Then, although Abcg1 deficiency protects mice from DIO, the exact role of Abcg1 in AT appears complex with a potential antagonist role of Abcg1 in AT, in promoting adipocyte maturation and fat mass growth on the one hand and in attenuating inflammation on the other hand. In this context, it is interesting to note that glucagon-like peptide 1 (GLP-1)-based therapy (vildagliptin and exendin-4) was able to induce the expression of Abcg1 and cholesterol efflux in mouse adipocytes [124].

### 8.2. Genetic Modulation of Human ABCG1

Several studies reported that human *ABCG1* was expressed in adipose tissue [77,80] and similar to what observed in mice, *ABCG1* expression was highly induced during adipocyte maturation [75]. No association between *ABCG1* SNPs and BMI or waist-to-hip ratio was observed in individuals with a BMI < 30 kg/m^2^ [65,67,73]. However, genotyping of two functional *ABCG1* SNPs (rs1378577, −134 T > G and rs1893590, −204 A > C in the promoter region) in 1320 French morbidly obese patients (BMI > 40 kg/m^2^) revealed that those two SNPs were associated with BMI after adjustment for diabetes and HOMA-IR [75]. Indeed, obese individuals homozygous for the frequent allele of each SNP (rs1378577, TT and rs1893590, AA) displayed the higher BMI. Also BMI increased in parallel with the amount of the frequent AT haplotype. In coherence with this result, obese individuals carrying the −134TT or −204AA genotype exhibited the higher fat mass index. Analysis of *ABCG1* expression in adipose tissue biopsies indicated that *ABCG1* mRNA levels were more elevated in adipose tissue from obese patients carrying the AT haplotype than those carrying the CG haplotype and *ABCG1* mRNA levels in adipose tissue were positively correlated to adipocyte diameter. Association of *ABCG1* genotype with obesity was replicated in two independent populations composed of either 595 severely obese (35 < BMI < 40 kg/m^2^) or 216 diabetic obese (30 < BMI < 35 kg/m^2^) from the diabetes atorvastatin lipid intervention (DALI) subjects [75]. This study reported for the first time that genetic modulation of *ABCG1* was intimately linked to fat mass growth and obesity in humans suggesting that ABCG1 might represent a potential therapeutic target in obesity [125]. Although this study suggests that an increase of ABCG1 expression in adipose tissue was positively associated with weight gain and BMI, it however appears that weight loss in obese individuals was not accompanied by a decrease of *ABCG1* expression in adipose tissue. Indeed, quantification of *ABCG1* mRNA levels in subcutaneous adipose tissue from obese individuals following caloric restriction [126,127,128,129] or surgery [130] indicated that *ABCG1* expression was not altered, the latter could even be increased [131].

### 8.3. Epigenetic Modulation of Human ABCG1

Although most of the metabolic disturbances in fat cells are normalized following gastric bypass surgery, patients in a post-obese (PO) state display adipose hyperplasia characterized by a smaller size but an increased number of fat cells as compared with never-obese (NO) women. In order to identify differentially methylated DNA sites linked to adipocyte hyperplasia in PO, genome-wide DNA methylation was analyzed in abdominal subcutaneous fat cells from women two years after gastric bypass surgery (PO, *n* = 16) and from never-obese women (NO, *n* = 14) [132]. Reduced DNA methylation at the *ABCG1* CpG site cg10192877 was detected in fat cells from PO women in comparison to NO women, which was associated with increased *ABCG1* mRNA levels in subcutaneous adipose tissue. Those results suggest that hyperplasia observed in PO women is associated with elevated ABCG1 expression in fat cells as compared NO women.

In order to decipher molecular mechanisms associated to obesity that contribute to obesity-related diseases, such as T2D and CVD, transcriptome and epigenome of circulating monocytes were conducted in 1264 participants from the multi-ethnic study of atherosclerosis (MESA) [133]. This study demonstrated that alterations of a cellular cholesterol metabolism network of 11 BMI-associated genes (including *ABCG1*) were associated with T2D and coronary artery calcium. More precisely, *ABCG1* expression in the coexpressed network module was downregulated with increasing BMI whereas methylation at the *ABCG1* CpG site cg06500161 was positively associated with BMI. Thus, the relationship between BMI and the expression of *ABCG1* in the module was partially explained through methylation [133]. Analysis of whole-genome DNA methylation in peripheral blood leucocytes from 2097 African American adults in the atherosclerosis risk in communities (ARIC) study reported that the increased methylation at two CpG sites within the *ABCG1* gene (cg06500161 and cg27243685) was positively associated with BMI [134]. Moreover, this study highlighted that methylation at cg06500161 was positively associated with waist circumference (WC). Finally, a very recent analysis carried out in a cohort of 1058 US women who had a sister with breast cancer but had not been diagnosed for the disease themselves (Sister Study) confirmed that blood DNA methylation of *ABCG1* (cg06500161) was associated with BMI [135]. Although the primary endpoint of those studies was T2D, a positive association between the *ABCG1* locus cg06500161 was reported with BMI in the Botnia prospective study [80], the LOLIPOP study [118] and with WC in the SAFHS [120]. Interestingly, the association of cg06500161 with obesity-related traits, such a BMI, waist-to-hip ratio and fat mass (total, android, ginoid and trunk) was only significant in the main group of individuals with BMI < 25 kg/m^2^ [118].

Taken together, results from studies in mice and in humans led to conflicting conclusions. Findings from Abcg1-deficient mice suggest that Abcg1 promotes diet-induced obesity through the contribution of adipocyte Abcg1 in adipogenesis and fat mass growth. The deleterious role of Abcg1 in obesity is supported by genetic modulation of *ABCG1* in populations of obese individuals, although analysis of adipose *ABCG1* expression following weight loss in both mice and humans appears in contradiction with the proposed model for ABCG1. On the contrary, epigenetic modulation of *ABCG1* was undoubtedly positively associated with an increased BMI and BMI-related traits which would support a protective role of ABCG1 in obesity. Those apparent contrasting observations clearly indicate that the role of adipose ABCG1 is still not yet fully elucidated and that further investigations are required in order to decipher the puzzling role of ABCG1 in obesity.

## 9. ABCG1: Role in Non-Alcoholic Fatty Liver Diseases (NAFLD)

### 9.1. Major Findings from Studies in Mice

Although no study aimed to investigate the contribution of Abcg1 in non-alcoholic steatohepatitis (NASH) and more widely in fatty liver diseases (NAFLD) has been conducted, several lines of evidence indicate that Abcg1 plays an important role in lipid homeostasis in the liver. In rat liver, most of the *Abcg1* expression resided in Kupffer cells (KC) (51% of total) whereas liver endothelial and parenchymal cells only accounted for 24% and 26% of total expression at the basal level. However a high-cholesterol diet highly induced *Abcg1* expression in parenchymal cells but not in KC and endothelial cells which brought the *Abcg1* expression in parenchymal cells up to 60% of total liver [136]. The use of Abcg1 deficient mice harboring a lacZ cassette insertion in exon 3 of the *Abcg1* locus confirmed that Abcg1 was expressed at very low levels in hepatocytes under a chow diet although stimulation with a LXR agonist (TO901317) led to significant increase of Abcg1 expression [55]. Nevertheless, the feeding of Abcg1-deficient mice with a high-cholesterol and high-fat diet led to a massive accumulation of lipids (TG, cholesterol ester and phospholipids) in the liver, in both parenchymal cells and KC, indicating that the very low levels of *Abcg1* mRNA in hepatocytes are critical in liver lipid homeostasis. By contrast, expression of the human ABCG1 transgene led to a reduction of both cholesterol ester and phospholipids in the liver from mice fed a high-cholesterol and high-fat diet as compared to wild-type mice [55]. In an independent study, a discrete increase in sterol biosynthetic intermediate levels (lathosterol, lanosterol and desmosterol) was observed in liver from mice expressing the human *ABCG1* transgene fed a high-fat and high-cholesterol diet in comparison to wild-type animals [61]. Moreover, an increase of the hepatic expression of genes controlling cholesterol and fatty acids synthesis was observed in *Abcg1*^−/−^ mice fed a chow diet with an increase of 3-hydroxy-3-methyl-glutaryl-coenzyme A reductase (*Hmgcr*), farnesyl pyrophosphate (*Fpp*), acetylCoA carboxylase (*Acc*), sterol-CoA desaturase (*Sdc1*) and *Srebp1c* mRNA levels in comparison to control mice. However, an opposite decrease of *Srebp2, Ldlr and Hmgcr* mRNA levels was equally reported in *Abcg1*^−/−^ mice fed a chow or a high-cholesterol diet relative to control mice, suggesting a decreased hepatic cholesterol synthesis and uptake in Abcg1-deficient mice [62]. In the latter study, feeding with a high-cholesterol diet led to a significant more elevated biliary cholesterol secretion in *Abcg1*^−/−^ mice compared with controls. Although Abcg1 was initially proposed to protect from lipid accumulation in the liver under a high-fat and high-cholesterol diet [55], Buchmann et al. unexpectedly reported that ablation of Abcg1 prevented the diet-induced lipid accumulation in liver from mice fed a high-fat diet devoid of cholesterol [109]. This opposite phenotype might result from the activation of the LXR pathway in cholesterol-enriched diets which in turn might stimulate lipogenesis and TG synthesis through SREBP-1c activation. However, the observation that liver TG were found decreased in *Abcg1*^−/−^ mice fed a chow diet supplemented with a LXR agonist (TO901317) as compared to control mice [62] does not support this hypothesis and suggests that a different mechanism occurs in order to explain the diet-specific control of liver lipid homeostasis by Abcg1.

### 9.2. Sparse Observations in Humans

Little information is available about the role of ABCG1 in lipid homeostasis in human liver. Analysis of expression of genes involved in cholesterol homeostasis in liver biopsies from obese patients that underwent bariatric surgery (14 with NASH and 17 with hepatosteatosis) in comparison to control liver biopsies from cadaveric liver donors or resection of liver metastasis (*n* = 7) indicated that liver *ABCG1* mRNA levels were modestly increased in patients with steatosis with or without NASH [137]. Interestingly, and in coherence with observation in Abcg1-defiencient mice fed a high-cholesterol diet [62], an increased expression of 3-hydroxy-3-methylglutaryl-CoA reductase (*HMGCR*) and sterol regulatory element-binding protein 2 (*SREBP2*) was also observed in liver from steatosis with or without NASH as compared to control biopsies [137]. However a more recent study performed in liver biopsies from 84 morbidly obese Mexican mestizo subjects who underwent bariatric surgery made the opposite observations [138]. Indeed, in this study, a reduction of liver ABCG1 protein levels was detected in patients with NASH as compared to control or steatosic livers while corresponding mRNA levels tended to be increased. Interestingly, ABCG1 protein levels were further decreased according to the different grades of fibrosis.

Thus, although ABCG1 appears to play an important role in the control of liver lipid homeostasis in mice in a diet-specific manner, such a role in humans remains to be demonstrated. Therefore, studies analyzing the genetic and epigenetic modulation of *ABCG1* in populations of patients with hepatosteatosis or NASH will greatly help to evaluate the contribution of ABCG1 expression in NAFLD.

## 10. Conclusions

A comprehensive review of the literature brought to light the critical role of the human ABCG1 transporter in CMD. Indeed, by redistributing cholesterol between cell membranes, ABCG1 not only maintains cellular cholesterol homeostasis but is also involved in the secretion and bioavailability of key molecules (LPL and insulin) controlling glucose and lipid metabolism. Studies in both mice and in humans, demonstrate modulation of ABCG1 expression in association with a large spectrum of metabolic disorders including diabetes, insulin resistance, obesity and CAD. However the precise role of ABCG1 in those disorders is still under debate since results obtained from studies in mice do not corroborate observations in humans and vice-versa. Nevertheless, it becomes clear that the epigenetic modulation of *ABCG1* is consistently associated with several metabolic disorders, including TG metabolism, diabetes-related traits, T2D risk, obesity and in a lesser degree to CAD (Table 2). Those studies highlight that methylation at the *ABCG1* in response to environmental exposure might be critical in CMD. Indeed, CMD are complex diseases that result from close interactions between genetic and epigenetic mechanisms which are greatly influenced by environmental stimuli. However, further studies are needed to elucidate mechanisms underlying such regulations and to determine whether ABCG1 might constitute a therapeutic target in CMD.

## Figures and Tables

**Table 1:** Association between methylation at different ATP-binding cassette G1 (ABCG1) CpG loci and plasma lipid levels. HDL, high-density lipoprotein; TG, triglycerides; KORA, cooperative health research in the region of Augsburg; InCHIANTI, aging in the chianti area; FHS, Framingham heart study; PIVUS, prospective investigation of the vasculature in Uppsala seniors; PPHT; postprandial hypertriglyceridemia; GOLDN, genetics of lipid lowering drugs and diet network.

Lipid Trait	CpG Site	Position	Direction	Cohort	*n*	*p* Value	Reference
**HDL**	**cg06500161**	43,656,588	−	KORA F3, KORA F4 and InCHIANTI (meta-analysis)	2747	9.00 × 10^−11^	[77]
			−	Rotterdam discovery/replication	725/760	9.5 × 10^−23^	[78]
			−	FHS and PIVUS	2306	1.2 × 10^−34^	[79]
**TG**	**cg06500161**	43,656,588	+	KORA F3, KORA F4 and InCHIANTI (meta-analysis)	2747	5.56 × 10^−10^	[77]
			+	Botnia prospective study	258	0.001	[80]
			+	Rotterdam discovery/replication	725/760	1.4 × 10^−24^	[78]
			+	FHS and PIVUS	2306	2.29 × 10^−48^	[79]
			+(PPHT)	GOLDN	979	4.25 × 10^−9^	[82]
	**cg27243685**	43,642,366	+	KORA F3, KORA F4 and InCHIANTI (meta-analysis)	2747	2.49 × 10^−5^	[77]
			+	FHS and PIVUS	2306	8.12 × 10^−26^	[79]
	**cg07397296**	42,235,165	+	KORA F3, KORA F4 and InCHIANTI (meta-analysis)	2747	3.78 × 10^−3^	[77]
	**cg01176028**	43,653,234	+	FHS and PIVUS	2306	5 × 10^−9^	[79]
	**CpGC3**	Not specified	+	Canadian familial Hypercholesterolemia	98	0.02	[81]

**Table 2:** Relation between methylation at different *ABCG1* CpG loci and cardiovascular diseases, prevalence and incidence of type 2 diabete and obesity. CHD, coronary heart diseases; T2D, type 2 diabetes; MI, myocardial infarction; KORA, cooperative health research in the region of Augsburg; InCHIANTI, aging in the Chianti area; HOMA-IR, homeostatic model assessment of insulin resistance; GOLDN, genetic of lipid lowering drugs and diet network; HbA1c, glycated haemoglobin; BMI, body mass index; LOLIPOP, London life science prospective population; HTGW, hypertriglyceridemic waist; WC, waist circumference; SAFHS, San Antonio family heart study; MESA, multi-ethnic study of atherosclerosis; ARIC, atherosclerosis risk in communities; FHS, Framingham heart study; PIVUS, prospective investigation of the vasculature in Uppsala seniors.

CpG Site	Position	CVD	Type 2 Diabete	Obesity	Direction	Cohort	*n*	Reference
**CpGC3**	*Not specified*	CAD			↔	Canadian Familial Hypercholesterolemia	22/22	[81]
**ABCG1 promoter**	*Not specified*	CHD			+	Chinese Han population	139	[99]
**cg06500161**	43,656,588	MI			+	KORA F3, KORA F4 and InCHIANTI (meta-analysis)	2747	[77]
			Fasting insulin and HOMA-IR		+	GOLDN	837	[116]
			Fasting glucose, fasting insulin, HbA1c and HOMA-IR		+	KORA F4	1440	[117]
			Risk of future T2D (fasting glucose, HbA1c, fasting insulin and HOMA-IR)	BMI and WHR, Fat mass	+	LOLIPOP	25,372	[118]
			Risk of future T2D (fasting glucose, HbA1c, fasting insulin and HOMA-IR)	BMI	+	Non-diabetic from Botnia prospective study	258	[80]
			Risk of future T2D (fasting glucose and HOMA-IR)		+	SAFHS	850	[119]
			HTGW and risk of T2D	WC	+			[120]
				BMI	+	MESA	1264	[133]
				BMI and WC	+	ARIC	2097	[134]
				BMI	+	Sister study	1058	[135]
**cg27243685**	43,642,366	CHD			+	FHS and PIVUS	2306	[79]
				BMI	+	ARIC	2097	[134]
**cg10192877**	43,641,690			Post obese women after by-pass surgery	−	Women after bypass surgery/Controls	16/14	[132]
**cg01881899**	43,652,704		Fasting insulin and HOMA-IR		+	GOLDN	837	[116]

## Abbreviations

ABC	ATP-Binding Cassette
ApoA-I	Apolipoprotein A-I
BMI	Body Mass Index
CAD	Coronary Artery Disease
CHD	Coronary Heart Disease
CMD	CardioMetabolic Disease
CpG	Cytosine Guanine dinucleotide
CVD	CardioVascular Diseases
EWAS	Epigenome-Wide Association Study
GWAS	Genome-Wide Association Study
HDL HOMA-IR	High-Density Lipoprotein HOmeostatic Model Assessment of Insulin Resistance
LDL	Low-Density Lipoprotein
LPL	Lipoprotein Lipase
LXR	Liver X Receptor
MI	Myocardial Infarction
SM	Sphingomyelin
SNP SREBP	Single Nucelotide Polymorphism Sterol Regulatory Element-Binding Protein
T2D	Type 2 Diabetes
TC	Total Cholesterol
TG	Triglycerides
WC	Waist Circumference
